# Impact of selective immune-cell depletion on growth of *Mycobacterium tuberculosis* (Mtb) in a whole-blood bactericidal activity (WBA) assay

**DOI:** 10.1371/journal.pone.0216616

**Published:** 2019-05-17

**Authors:** Gail B. Cross, Benjamin C-M Yeo, Paul Edward Hutchinson, Mark C. Tan, Rupangi Verma, Qingshu Lu, Nicholas I. Paton

**Affiliations:** 1 Department of Medicine, Yong Loo Lin School of Medicine, National University of Singapore, Singapore; 2 Singapore Clinical Research Institute, Singapore; 3 Division of Infectious Diseases, University Medicine Cluster, National University Hospital, Singapore; 4 Flow Cytometry Lab, Life Sciences Institute, National University of Singapore, Singapore; Institut de Pharmacologie et de Biologie Structurale, FRANCE

## Abstract

We investigated the contribution of host immune cells to bacterial killing in a whole-blood bactericidal activity (WBA) assay, an ex vivo model used to test efficacy of drugs against *mycobacterium tuberculosis* (Mtb). We performed WBA assays with immuno-magnetic depletion of specific cell types, in the presence or absence of rifampicin. Innate immune cells decreased Mtb growth in absence of drug, but appeared to diminish the cidal activity of rifampicin, possibly attributable to intracellular bacterial sequestration. Adaptive immune cells had no effect with or without drug. The WBA assay may have potential for testing adjunctive host-directed therapies acting on phagocytic cells.

## Introduction

A variety of drugs (old/ re-purposed and new) with potential anti-mycobacterial activity require testing alone and in combinations, but there are limited options for initial screening in humans prior to launching expensive and laborious clinical trials [1]. There is also increasing interest in drugs that modulate the host immune response to mycobacterium tuberculosis (Mtb), which survives as a successful intracellular pathogen by evading host immune defence mechanisms. Approaches for initial testing of such host-directed therapies for TB are not established. The whole-blood bactericidal activity (WBA) assay is an established *ex vivo* model which tests the anti-mycobacterial efficacy of drugs, in the context of an additional contribution made by host immune cells found in whole blood [2–4]. Whilst this model has a theoretical advantage over testing in cell-free assays, the nature of the contribution of the host cells in controlling Mtb growth, with and without antibacterial drugs, has not been well described [5]. We performed this study to determine the magnitude of the contribution of immune cells to measured WBA in this assay.

## Materials and methods

Eight healthy volunteers were recruited over a five-month period from December 2016 to May 2017. WBA assays were performed on blood drawn (63ml, taken in sodium-heparin tubes) on the morning of the study day. Each sample was depleted of cells, in separate parallel experiments, using a magnetic platform (STEMCELL EasySep, STEMCELL Technologies Singapore, Pte Ltd, Singapore) and specific monoclonal antibodies against monocytes (CD14+), neutrophils (CD66b+, CD15+), natural killer (NK) (CD56+), CD4+ T cells, CD8+ T cells, B (CD19+) and dendritic cells (DCs) (CD11c+, CD123+). The extent of depletion was measured by flow cytometry. Fluorochrome-conjugated antibodies (S1 Table) were added to 100ul whole blood and incubated for 20 minutes before red blood cell lysis using FACS lysis solution (BD Biosciences). Cells were then washed twice with PBS and resuspended in FACS buffer (PBS + 0.2% BSA) before data acquisition. To determine depletion purity, staining panels were designed to detect specific populations of immune cell subsets in whole blood using the LSR Fortessa (BD Biosciences) for data acquisition and FlowJo (Treestar) for data analysis. Samples were first gated for single cells, then by size and by granularity using forward scatter and side-scatter. Finally to target the cell subtype a gating strategy was employed looking specifically at the listed cell differentiation markers (S2 Table). Samples with less than 75% depletion were not included in further analyses.

The WBA assay was performed as described in detail previously [2, 3, 6]. In brief, a standard stock was made for all experiments using Mycobacterium tuberculosis (H37Rv) grown in 7H9 medium to mid-log phase, and a standard curve was generated that related volume of that stock to the time to positivity (TTP) in culture in the MGIT960 detection system (Becton Dickinson, Franklin Lakes, USA). The volume of mycobacterial stock calculated from the standard curve to give a TTP of 5.5 days (0.5μL) was added to 300μL of the test blood sample (control without cell depletion, or sample following specific cell depletion) and topped up with sufficient tissue culture medium (RPMI-GlutaMAX) to make up total culture volume of 600μL. Rifampicin was added in half of the samples in each cell depletion group to make up a total concentration of 1ug/ml (approximating the levels seen in plasma approximately 1 hour after a standard oral dose of 10mg/kg of rifampicin, representing about 25–50% peak WBA of the drug; level selected to avoid dominating the contribution of the immune cells to bacterial control).

Cultures were incubated at 37°C for 72 hours, then the liquid phase was removed, the cells were lysed, and the pellet re-suspended and inoculated into MGIT tubes. The TTP was recorded (to the nearest minute). WBA cultures were set-up in duplicate for each experimental condition and the mean TTP calculated. Control cultures (in duplicate) were set-up on the same day by inoculating the standard volume of stock directly into MGIT tubes. The WBA at each of the individual cell depletion experiments was obtained from the difference between the log of the volume on the standard curve that corresponded to the TTP for that cell depletion experiment and the log of the volume corresponding to the TTP of the control culture. This is equivalent to the difference in log of bacterial colony forming units (CFU) between the sample and the control, reported as ΔlogCFU.

Participants also had an interferon gamma release assay (IGRA) (QuantiFERON–TB Gold (QFT), Qiagen, Hilden, Germany) performed and participants were classified as positive or negative based on the manufacturer’s recommendations. The study was approved by the National Healthcare Group Domain Specific Review Board (Singapore) and volunteers gave written informed consent.

## Results

Cell depletion was successful (>75% depletion) in 4–8 assays for all cell types (median depletion 94.4%, range 75.2–100%; S3 Table) except for CD15+ neutrophils and CD123+ DCs (median depletion 25.8% and 35.1% respectively; these assays were not analysed further).

**Fig 1:**
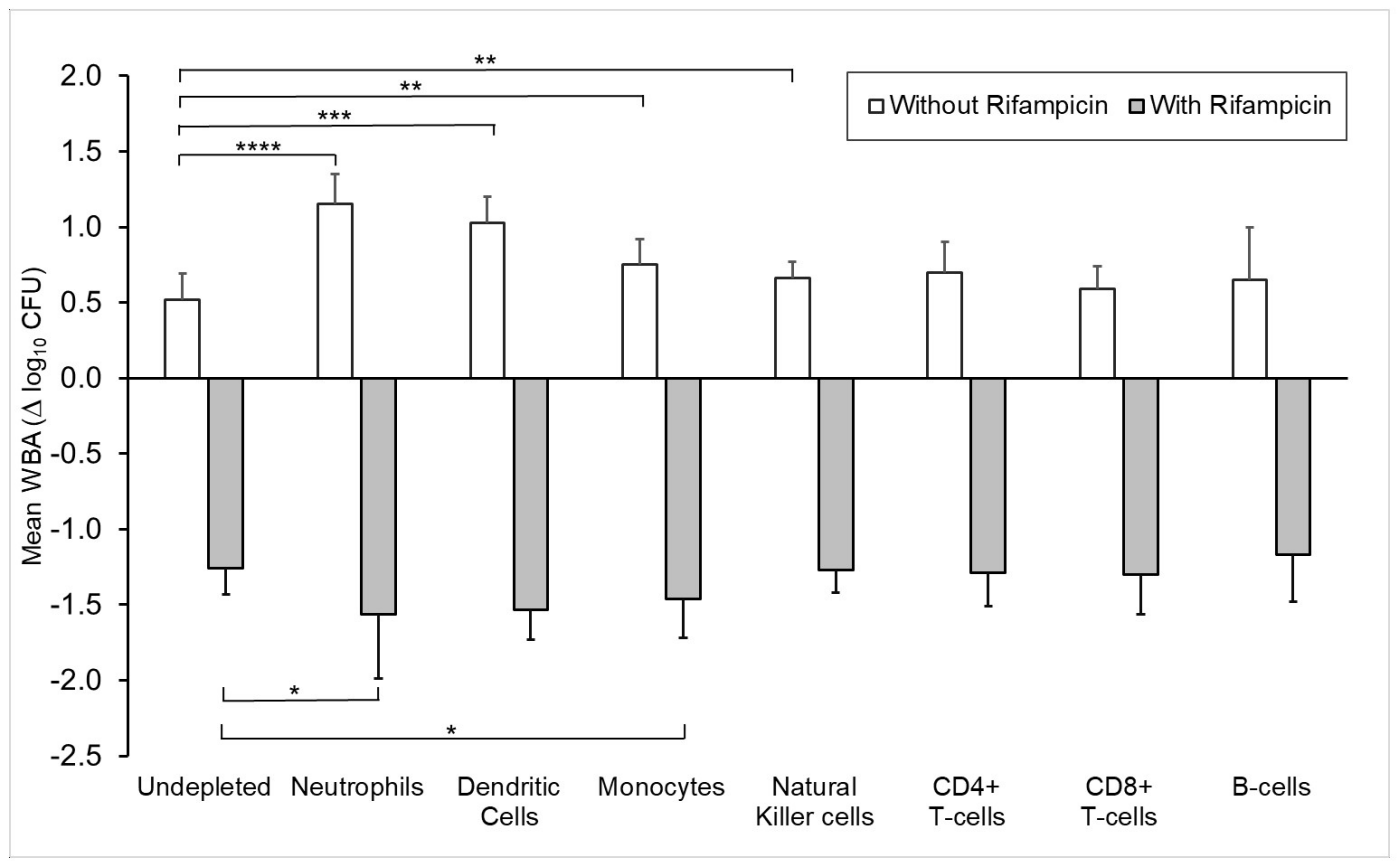
Effect of selective cell subtype depletion on Mtb growth in the presence or absence of low-dose rifampicin. Neutrophils are CD66b+ cells. Dendritic cells are CD11c+ cells. WBA values were compared between undepleted culture and selective cell depletion culture using a paired-sample t-test. Error bar represents one SD. * for < 0.05, ** for < 0.01, *** for < 0.001, **** for < 0.0001.

In the absence of rifampicin, depletion of CD66b+ neutrophils, CD11c+ DCs, monocytes and NK cells increased the rate of growth of Mtb compared to un-depleted whole blood (Fig 1). The largest effect was seen with depletion of neutrophils. There was also a trend to increased Mtb growth with depletion of adaptive immune cells (CD4+ T-cells and CD19+ B-cells) but this did not reach statistical significance (Fig 1 and S4 Table).

In the presence of low-dose (1ug/ml) rifampicin, depletion of neutrophils and monocytes decreased the rate of growth compared to the un-depleted whole blood (i.e. enhanced drug-related bactericidal activity) (Fig 1). Depletion of DCs also showed a trend to reducing the rate of growth of Mtb. Depletion NK cells, CD4+ T-cells and CD8+ T-cells and B cells did not have a significant impact (Fig 1 and S4 Table).

There was no significant difference in growth in any of the WBA experimental conditions comparing assay results for volunteers who were IGRA positive or negative (4 and 3 respectively; 1 not determined) (S1 Fig).

## Discussion

We found that immune cells influence mycobacterial growth in the WBA assay, although the relationship is complex, varying by cell type and by the presence or absence of rifampicin. Without rifampicin, innate immune cells controlled Mtb growth, with neutrophils having the greatest impact (i.e. their depletion lead to the greatest enhancement of growth). In human tuberculosis, neutrophils are the most abundantly infected phagocytic cell in respiratory samples [7]. The depletion of neutrophils from ex vivo whole blood has previously been found to enhance the growth of Mtb, and as in our study, neutrophilic depletion had a greater impact on Mtb growth than the depletion of other immune cell subtypes [8, 9]. However the role neutrophils play in TB disease is complex and has been associated with “failed immunity” and the excessive pathology of tuberculosis [10, 11]. Whilst neutrophils may act to control early infection through antigen presentation and the ensuing initiation of adaptive immunity, neutrophils undergo necrotic cell death brought about by virulent Mtb infection, are phagocytosed by macrophages, and it has previously been shown in a whole blood model that it is this presence of necrotic neutrophilic debris (as opposed to intact neutrophils) which propagate and enhance Mtb replication [9, 10, 12]. CD11c+ DCs, having differentiated from inflammatory circulating monocytes, have been shown to enter the lung parenchyma where they acquire then transport intact Mtb to draining lymph nodes, where they have a well-established role in initiating adaptive immunity [13–15]. The corollary of this study’s finding of increased Mtb growth in the absence of myeloid (CD11c+) DCs is that DCs in whole blood result in the reduced survival of Mtb. This has been previously shown in cell culture assays, but not in a whole blood culture model [16, 17]. Circulating monocytes were shown to have modest effects on controlling replication. This is consistent with the finding that monocytes themselves may not have a significant function in the control of Mtb, however their function of differentiating into tissue-resident macrophages and DCs in vivo contributes significantly to the control of Mtb [14, 18, 19]. Consistent with our findings that NK cells can control Mtb in WBA, the depletion of NK cells in PBMCs taken from blood of healthy TB exposed individuals, and depletion of the cell type in mice post BCG vaccination resulted in an increased Mtb burden, an impact likely to have been driven by the effect NK cells have on specific T cell responses against Mtb [20, 21]. Additionally recent evidence shows that despite being categorised as part of the innate immune system, NK cells have memory (after vaccination or after prior Mtb exposure), and these memory-NK cells can expand to provide protection against Mtb infection[22, 23]. In our study there was no difference seen between IGRA positive and negative individuals, however the numbers of participants were small and therefore not adequately powered to investigate this possibility.

Our findings agree with a previous WBA study where the depletion of CD4+ or CD8+ T-cells individually or together had no impact on control of virulent strains of Mtb (although depletion did have an effect on control of an attenuated stain) [24]. In another WBA study CD4+ T-cells depletion was shown to have affected the growth of the less virulent BCG organism, and this too only in tuberculin positive children and not those who were tuberculin negative [25]. We saw no difference in IGRA positive and negative individuals in our study.

Our results suggest that neutrophils and monocytes limit the bactericidal activity of rifampicin in the WBA assay (cell depletion increased the observed bactericidal activity of rifampicin). This may seem paradoxical given the observation that these cells controlled bacterial growth in the absence of rifampicin. A possible explanation is that these innate immune cells phagocytose Mtb and thereby protect from exposure to cidal levels of drug; or the intracellular environment induces the bacteria to enter a dormant state, with less metabolic activity and greater drug tolerance [26, 27].

Our findings support the belief that the WBA assay measures, at least in part, the sterilising activity of drugs against dormant, intracellular bacteria [28]. This could be an advantage compared to the standard early bactericidal activity (EBA) paradigm, currently used as the initial screen for cidal activity of new TB drugs and which measures predominantly extracellular drug activity [29]. Furthermore the assay may have the potential to assess drugs which target host immune pathways, an area of increasing interest [1]. In particular WBA might be a good platform to test drugs which affect immuno-metabolic pathways involved in autophagy, which is a key mechanism through which phagocytic cells induce intracellular killing of Mtb [30]. Whole-blood mycobacterial growth-inhibition assays (MGIA) such as WBA are also a good paradigm for testing the functional efficacy of Mtb vaccination and have been applied in this way in one clinical trial [5, 31, 32]. The most obvious limitation of the WBA paradigm is the uncertainty over which findings translate to in vivo outcomes. Key cell types for TB control in vivo, such as macrophages (long considered to be the primary niche for Mtb residence and replication), MAIT (mucosal associated invariant T) cells and γδ-T cells are scarce in peripheral blood [33–35] and the immune response in WBA is not representative of immune response at a tissue level [18].

The limitations of this study are the small sample size (although it was sufficient to demonstrate significant effects of depletion of some cell types); the lack of power to examine effect modification by IGRA status; the inability to deplete all cell types known to be relevant for TB control; and that we examined depletion of single cell types but not multiple cell-type depletions. Nevertheless, we found evidence to indicate that immune cells make an important contribution to cidal activity in the WBA assay. This assay may therefore be of value as an initial screening test for putative host-directed therapy drugs prior to their evaluation in definitive clinical trials.

## Supporting information

S1 Table Fluorochrome antibodies used by cell subset panel. (DOCX)

S2 Table Gating strategy utilised. (DOCX)

S3 Table Median cell depletion by cell type as measured by flow cytometry. (DOCX)

S4 Table WBA values of MTB growth in selective cell subtype depletion experiments. Values are mean (SD), and mean difference (95% CI). ^a^n is the number of observations where cell depletion was ≥ 75%. ^b^Mean WBA is the change in log colony forming units (Δlog CFU). ^c^Mean difference is the difference in growth (log_10_ CFU over the 72-hour incubation period) of a specific cell depletion culture minus the control culture with no cell depletion performed. ^d^WBA values were compared between undepleted culture and selective cell depletion culture using a paired-sample t-test. ^e^ CD66b+ Neutrophils. ^f^ CD11c+ Dendritic cells.
(DOCX)

S1 Fig Mean change of WBA of selective cell depletion culture from undepleted culture by QuantiFERON–TB Gold status, with/without rifampicin. Neutrophils are CD66b+ cells. Dendritic cells are CD11c+ cells. Height of the bars represent the mean change of WBA of selective cell depletion culture from undepleted culture. Error bar represents one SD.
(TIF)
