# Supplementary material for: Impact of selective immune-cell depletion on growth of Mycobacterium tuberculosis (Mtb) in a whole-blood bactericidal activity (WBA) assay
Source: PLoS One. 2019 May 17;14(5):e0216616. doi: 10.1371/journal.pone.0216616 (PMC6524797; doi:10.1371/journal.pone.0216616)
Supplement: S1 Table — (DOCX) [file pone.0216616.s001.docx]

**S1 Table. Fluorochrome antibodies used by cell subset panel.**

| **Cell Subset Panel** | **Antibody** | **Colour** | **Manufacturer** |
| --- | --- | --- | --- |
| **T- cell,**  **B-cell** | anti-CD45 | PerCP-Cy5.5, clone 2D1 | Biolegend |
|  | anti-CD3 | FITC, clone SK7 | Biolegend |
|  | anti-CD4 | Brillant Violet 605, clone OKT4 | Biolegend |
|  | anti-CD8a | Pacific Blue, clone HIT8a | Biolegend |
|  | anti-CD19 | APC, clone HIB19; B | Biolegend |
| **NK cells, neutrophils, monocytes** | anti-CD45 | PerCP-Cy5.5, clone 2D1 | Biolegend |
|  | anti-CD3 | FITC, clone SK7 | Biolegend |
|  | anti-CD14 | Pacific Blue, M5E2 | Biolegend |
|  | anti-CD16 | APC-Cy7, clone 3G8 | Biolegend |
|  | anti-CD56 | APC, clone HCD56 | Biolegend |
| **DCs** | anti-CD45 | PerCP-Cy5.5, clone 2D1 | Biolegend |
|  | anti-CD3 | FITC, clone SK7 | Biolegend |
|  | anti-CD19 | APC, clone HIB19 | Biolegend |
|  | anti-CD56 | APC, clone HCD56 | Biolegend |
|  | anti-CD14 | Pacific Blue, M5E2 | Biolegend |
|  | anti-HLA-DR | Brilliant Violet 650, clone L243 | Biolegend |
|  | anti-CD123 | PE-Cy7, clone 6H6 | eBioscience |
|  | anti-CD11c | PE, clone 3.9 | eBioscience |
