# Supplementary material for: Impact of selective immune-cell depletion on growth of Mycobacterium tuberculosis (Mtb) in a whole-blood bactericidal activity (WBA) assay
Source: PLoS One. 2019 May 17;14(5):e0216616. doi: 10.1371/journal.pone.0216616 (PMC6524797; doi:10.1371/journal.pone.0216616)
Supplement: S2 Table — (DOCX) [file pone.0216616.s002.docx]

**S2 Table. Gating Strategy utilised.**

| **Target Cell Subtype** | **Gating Strategy** |
| --- | --- |
| CD4+ T-cells | CD45^+^CD3^+^CD4^+^ |
| CD8+ T-cells | CD45^+^CD3^+^CD8^+^ |
| CD19+ B-cells | CD45^+^CD3^-^CD19^+^ |
| CD66b+ Neutrophils | CD45^+^CD16^+^ |
| CD15+ Neutrophils | CD45^+^CD16^+^ |
| CD14+ Monocytes | CD45^+^CD14^+^CD16^+^ |
| CD56+ Natural killer cells | CD45^+^CD3^-^CD56^+^ |
| CD123+ dendritic cells | CD45^+^lineage^-^HLA-DR^+^CD123^+^ |
| CD11c+ dendritic cells | CD45^+^lineage^-^HLA-DR^+^CD11c^+^ |

Lineage refers to CD3+CD14+CD19+CD56+ cells.
