# Supplementary material for: Impact of selective immune-cell depletion on growth of Mycobacterium tuberculosis (Mtb) in a whole-blood bactericidal activity (WBA) assay
Source: PLoS One. 2019 May 17;14(5):e0216616. doi: 10.1371/journal.pone.0216616 (PMC6524797; doi:10.1371/journal.pone.0216616)
Supplement: S3 Table — (DOCX) [file pone.0216616.s003.docx]

**S3 Table. Median cell depletion by cell type as measured by flow cytometry**.

| **Cell type** | **Observations** | **Depletion (%)** | **Range** |
| --- | --- | --- | --- |
| CD66b Neutrophils | n=8 | 96.4 | 84.0, 99.9 |
| CD11c Dendritic Cells | n=4 | 92.9 | 75.2, 100.0 |
| CD14 Monocytes | n=8 | 94.4 | 78.8, 99.2 |
| CD56 NK Cells | n=6 | 98.3 | 97.2, 100.0 |
| CD4 T Cells | n=6 | 98.9 | 80.7, 99.1 |
| CD8 T Cells | n=6 | 92.3 | 89.8, 98.8 |
| CD19 B Cells | n=5 | 88.2 | 81.4, 93.3 |

Values are the percentage of each cell subtype depleted by the procedure compared to the undepleted whole blood sample from the same volunteer. Individual samples with <75% cells depleted were excluded from the analysis groups. CD15+ neutrophil depletion assays (median depletion 25.8%, range 12% to 71.7%) and CD123+ DC depletion assays (median depletion 35.1%, range -4.8% to 71.3%) were excluded entirely from further analyses.
