# Supplementary material for: Impact of selective immune-cell depletion on growth of Mycobacterium tuberculosis (Mtb) in a whole-blood bactericidal activity (WBA) assay
Source: PLoS One. 2019 May 17;14(5):e0216616. doi: 10.1371/journal.pone.0216616 (PMC6524797; doi:10.1371/journal.pone.0216616)
Supplement: S4 Table — Values are mean (SD), and mean difference (95% CI). an is the number of observations where cell depletion was ≥ 75%. bMean WBA is the change in log colony forming units (Δlog CFU). cMean difference is the difference in growth (log10 CFU over the 72-hour incubation period) of a specific cell depletion culture minus the control culture with no cell depletion performed. dWBA values were compared between undepleted culture and selective cell depletion culture using a paired-sample t-test. e CD66b+ Neutrophils. f CD11c+ Dendritic cells. (DOCX) [file pone.0216616.s004.docx]

**S4 Table.** **WBA values of MTB growth in selective cell subtype depletion experiments.**

| **Cell type** | **n^a^** | **WITHOUT RIFAMPICIN** | | | **WITH RIFAMPICIN** | | |
| --- | --- | --- | --- | --- | --- | --- | --- |
|  |  | **WBA (SD)^b^** | **Difference (95% CI)^c^** | **p value^d^** | **WBA (SD) ^b^** | **Difference (95% CI) ^c^** | **p value ^d^** |
| Undepleted | 8 | 0.52 (0.17) |  |  | -1.26 (0.17) |  |  |
| Neutrophils^e^ | 8 | 1.15 (0.20) | 0.62 (0.45, 0.80) | <0.0001 | -1.56 (0.43) | -0.30 (-0.59, -0.02) | 0.0416 |
| Dendritic Cells^f^ | 4 | 1.03 (0.17) | 0.47 (0.39, 0.54) | 0.0003 | -1.53 (0.20) | -0.14 (-0.35, 0.08) | 0.1327 |
| Monocytes | 8 | 0.75 (0.17) | 0.23 (0.10, 0.35) | 0.0033 | -1.46 (0.26) | -0.20 (-0.35, -0.04) | 0.0182 |
| Natural Killer cells | 6 | 0.66 (0.11) | 0.16 (0.07, 0.26) | 0.0069 | -1.27 (0.15) | -0.05 (-0.25, 0.14) | 0.5112 |
| CD4+ T-cells | 6 | 0.70 (0.20) | 0.20 (-0.07, 0.48) | 0.1187 | -1.29 (0.22) | 0.01 (-0,11, 0.13) | 0.8163 |
| CD8+ T-cells | 6 | 0.59 (0.15) | 0.09 (-0.05, 0.23) | 0.1454 | -1.30 (0.26) | 0.00 (-0.12, 0.13) | 0.9817 |

Values are mean (Standard Deviation), and mean difference ( 95% Confidence interval).

^a^n is the number of observations where cell depletion was ≥ 75%.

^b^Mean WBA is the change in log colony forming units (Δlog CFU).

^c^Mean difference is the difference in growth (log_10_ CFU over the 72-hour incubation period) of a specific cell depletion culture minus the control culture with no cell depletion performed.

^d^WBA values were compared between undepleted culture and selective cell depletion culture using a paired-sample t-test.

^e^ CD66b+ Neutrophils.

^f^ CD11c+ Dendritic cells.
