# Supplementary figures and images for: Impact of selective immune-cell depletion on growth of Mycobacterium tuberculosis (Mtb) in a whole-blood bactericidal activity (WBA) assay
Source: PLoS One. 2019 May 17;14(5):e0216616. doi: 10.1371/journal.pone.0216616 (PMC6524797; doi:10.1371/journal.pone.0216616)

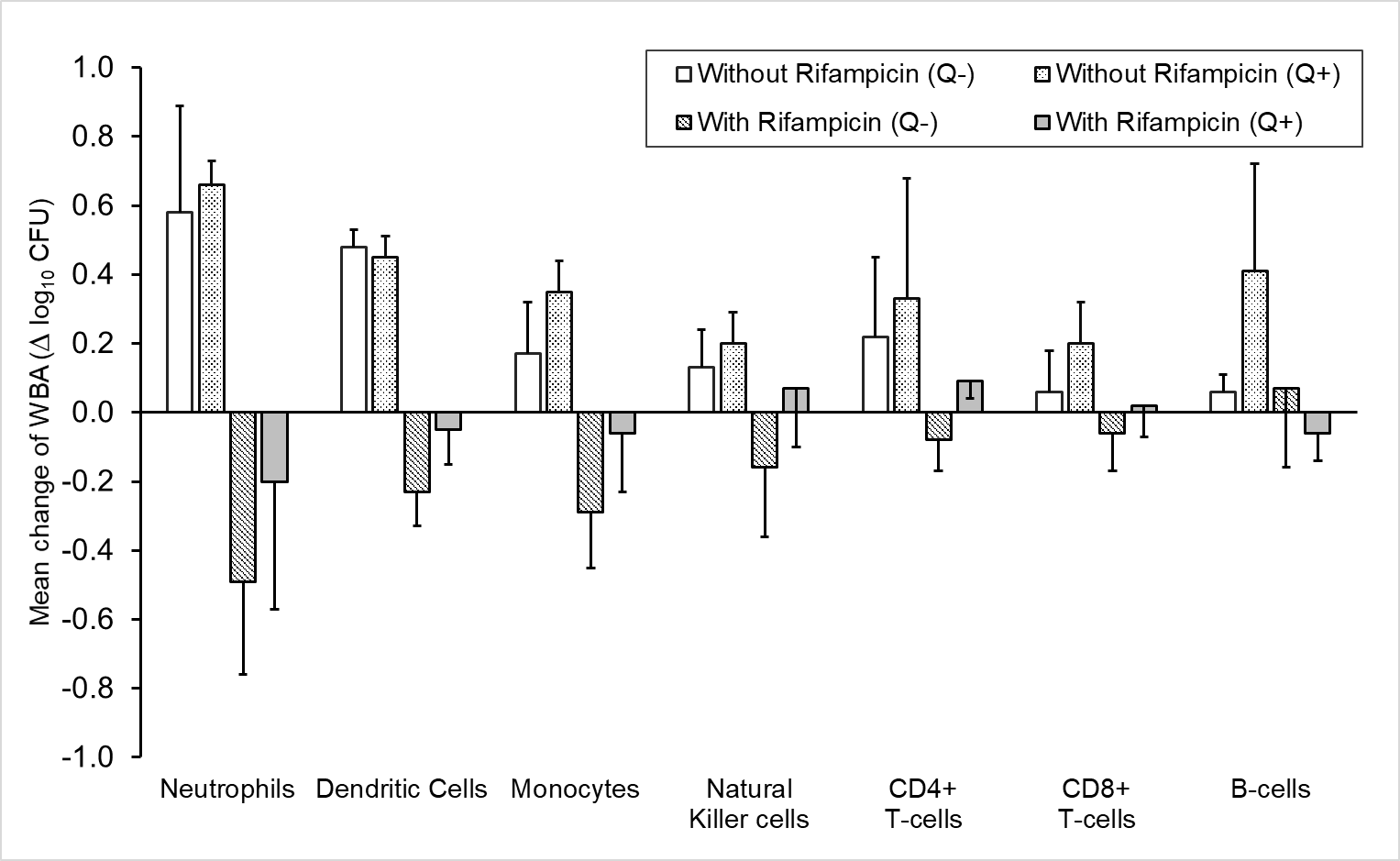

Supplement: S1 Fig — Neutrophils are CD66b+ cells. Dendritic cells are CD11c+ cells. Height of the bars represent the mean change of WBA of selective cell depletion culture from undepleted culture. Error bar represents one SD. (TIF) [file pone.0216616.s005.tif]
